# *Trypanosoma cruzi*-specific CD8^+^ T cells and other immunological hallmarks in chronic Chagas cardiomyopathy: Two decades of research

**DOI:** 10.3389/fcimb.2022.1075717

**Published:** 2023-01-04

**Authors:** Concepción J. Puerta, Adriana Cuellar, Paola Lasso, Jose Mateus, John M. Gonzalez

**Affiliations:** ^1^ Laboratory of Molecular Parasitology, Infectious Diseases Group, Department of Microbiology, School of Sciences, Pontificia Universidad Javeriana, Bogotá, Colombia; ^2^ Clinical Laboratory Sciences Group, Department of Microbiology, School of Sciences, Pontificia Universidad Javeriana, Bogotá, Colombia; ^3^ Group of Biomedical Sciences, School of Medicine, Universidad de Los Andes, Bogotá, Colombia

**Keywords:** CD8^+^ T cell, Chagas disease, *Trypanosoma cruzi*, immune response, Chagas cardiomyopathy, TcTLE peptide

## Abstract

*Trypanosoma cruzi*, the causal agent of Chagas disease, has coexisted with humans for thousands of years. Therefore, the parasite has developed several mechanisms of antigenic variability that has allowed it to live inside the cells and evade the host immune response. Since *T. cruzi* displays an intracellular cycle-stage, our research team focused on providing insights into the CD8^+^ T cells immune response in chronic Chagas cardiomyopathy. We began our work in the 2000s studying parasite antigens that induce natural immune responses such as the KMP11 protein and TcTLE, its N-terminal derived peptide. Different approaches allowed us to reveal TcTLE peptide as a promiscuous CD8^+^ T cell epitope, able of inducing multifunctional cellular immune responses and eliciting a humoral response capable of decreasing parasite movement and infective capacity. Next, we demonstrated that as the disease progresses, total CD8^+^ T cells display a dysfunctional state characterized by a prolonged hyper-activation state along with an increase of inhibitory receptors (2B4, CD160, PD-1, TIM-3, CTLA-4) expression, an increase of specific terminal effector T cells (T_TE_), a decrease of proliferative capacity, a decrease of stem cell memory (T_SCM_) frequency, and a decrease of CD28 and CD3ζ expression. Thus, parasite-specific CD8^+^ T cells undergo clonal exhaustion, distinguished by an increase in late-differentiated cells, a mono-functional response, and enhanced expression of inhibitory receptors. Finally, it was found that anti-parasitic treatment induces an improved CD8^+^ T cell response in asymptomatic individuals, and a mouse animal model led us to establish a correlation between the quality of the CD8^+^ T cell responses and the outcome of chronic infection. In the future, using OMICs strategies, the identification of the specific cellular signals involved in disease progression will provide an invaluable resource for discovering new biomarkers of progression or new vaccine and immunotherapy strategies. Also, the inclusion of the TcTLE peptide in the rational design of epitope-based vaccines, the development of immunotherapy strategies using T_SCM_ or the blocking of inhibitory receptors, and the use of the CD8^+^ T cell response quality to follow treatments, immunotherapies or vaccines, all are alternatives than could be explored in the fight against Chagas disease.

## 1 Introduction

Chagas disease is caused by the protozoan parasite *Trypanosoma cruzi.* This disease is endemic in 21 Latin American countries and has been exported to nonendemic regions on five continents by human migrations, constituting a global health problem. The disease is classified by the World Health Organization (WHO) as one of the 20 Neglected Tropical Diseases (NTDs) (WHO, 2017; WHO, 2022). Chagas disease has a socioeconomic burden 7.5 times greater than that generated by other parasitic infection such as malaria (Bern, 2015). Currently, the disease affects more than 6 million people in Latin America with 12,000 deaths, 30,000 new cases, and 8,600 new-born infections per year, and 70 million people at risk of contracting the infection PAHO, 2022. It is estimated that there are 300,000 infected individuals in the United States (USA) and approximately 59,000 to 108,000 infected individuals in Europe (Pérez-Molina and Molina, 2018).

The main transmission route of *T. cruzi* in endemic countries is through triatomine insects belonging to the family *Reduviidae*, which generally colonize rural homes built with bahareque walls and palm roofs. The disease is linked to poverty, and patients must overcome multiple barriers to access both diagnosis and treatment. Conversely, in non-endemic countries, the most common transmission forms are *via* congenital, transfusion, or organ transplant infections (Rosas et al., 2012). Therefore, despite the progress made in the interruption of vector transmission in some municipalities and countries in Latin America, the WHO calls on affected governments and the scientific community to work together to achieve control of this disease (WHO, 2017; WHO, 2022).

Chagas disease is highly disabling, affecting the quality of life not only of the patient but also of the entire family nucleus and influencing also the productivity, besides the economy of the countries where this disease is present. The global costs of health and disability-adjusted life years (DALYs) are approximately USD 7.19 billion per year and $188.80 billion per life, respectively; exceeding the burden of diseases such as acute diarrhoeal caused by rotavirus or cervical cancer (Lee et al., 2013). Remarkably, more than 10% of these costs come from nonendemic countries such as the USA and Canada (Lee et al., 2013).

Chagas disease occurs with two clinical phases: acute and chronic. Acute disease is more common in endemic regions, particularly in children, who may present a fever indistinguishable from other tropical infectious diseases. In some vector infection cases, characteristic signs such as chagoma or Romaña’s sign can occur. During the acute phase, high levels of parasitemia induce a strong innate and adaptive immune response that controls but does not eliminate the parasite (López et al., 2011). In 70-75% of cases, the chronic phase is characterized by being asymptomatic or indeterminate. However, 25-30% of individuals progress in years or even decades to the determined or symptomatic phase, with the vast majority presenting cardiac damage and, to a lesser extent, digestive compromise, mainly in the colon or esophagus (Rosas et al., 2012). Chronic Chagas cardiomyopathy (CCC) is the most important clinical manifestation of the disease due to its severity, frequency, morbidity, and lethality (Tanowitz et al., 2015).

The parasite can persist in tissues and cause cellular damage increasing the inflammatory response (Rosas et al., 2012; Klahr et al., 2016). In patients with CCC, the parasite tissue persistence has been demonstrated using molecular biology techniques (Sabino et al., 2015). Importantly, this parasite tissue persistence is accompanied by the presence of cellular infiltrate, mostly CD8^+^ and CD4^+^ T cells, macrophages, and to a lesser extent, B cells (Argüello et al., 2014). Indeed, the immune response and the balance between the effector and the regulatory mechanisms seem to be determinants in the outcome of the disease.

Although Chagas disease was discovered more than 100 years ago by Carlos Chagas in Brazil, to date, there are no biomarkers that indicate the progression of cardiac disease from the asymptomatic to the symptomatic chronic phase, and no vaccines have been developed. Regarding treatment, there are only two specific antiparasitic drugs available. Benznidazole and Nifurtimox are indicated in patients with the disease during the acute and asymptomatic phases, while in chronically infected patients with cardiac damage, parasitemia control does not improve the disease symptoms or progression (Morillo et al., 2015).

**Figure 1:**
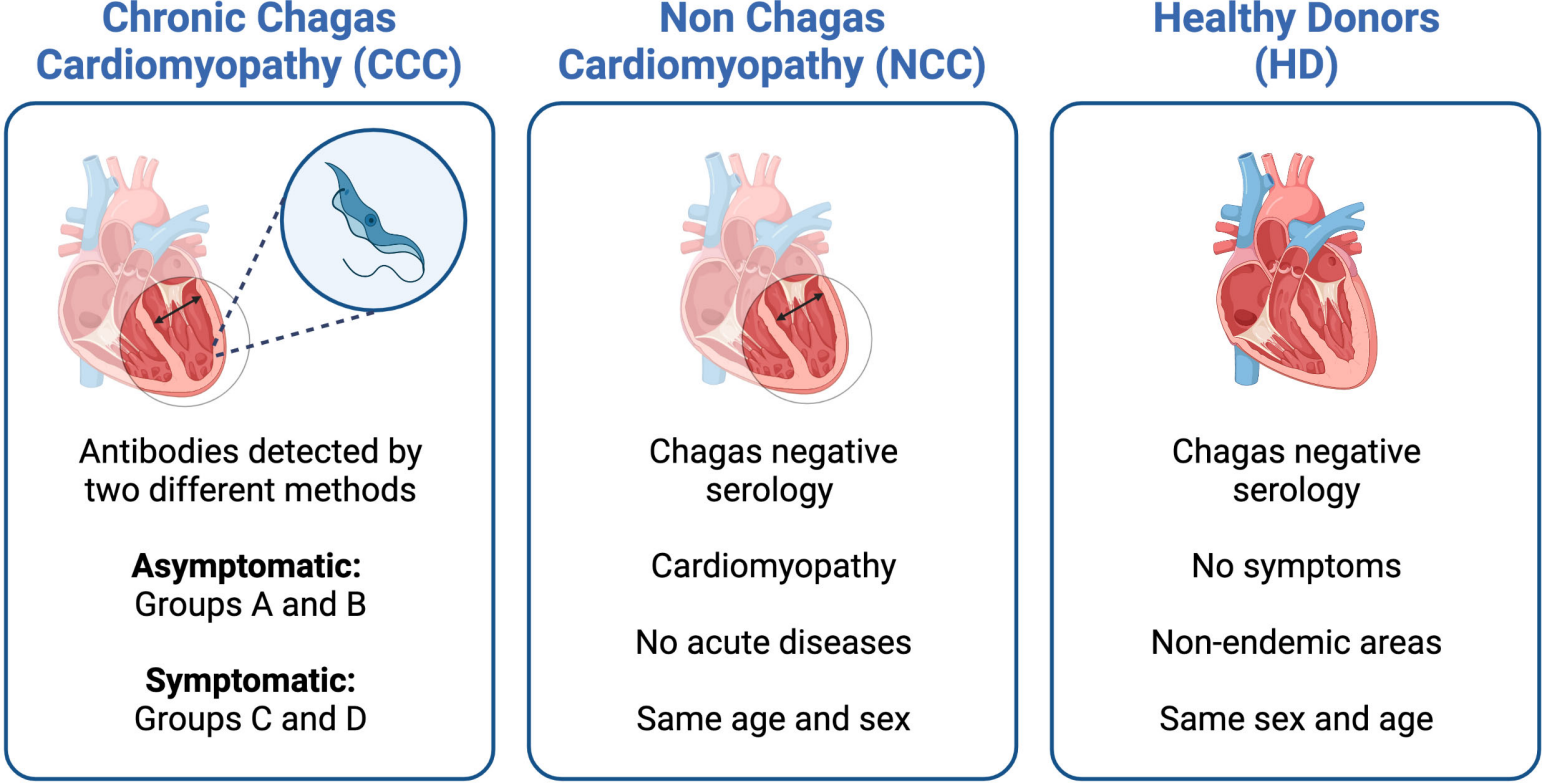
Characteristics of the groups of patients included in the different studies. Serology was used to determine IgG anti-*Trypanosoma cruzi-specific* antibodies.

As *T. cruzi* is an intracellular pathogen, our research group focused its efforts on elucidating the immune response of CD8^+^ T cells, starting with studying *T. cruzi* antigens capable of inducing an immune response during natural infection. We continued with research on the phenotypic and functional characteristics of the total and parasite-specific T cell responses to finally establish the correlation of these cell populations with protection in anti-parasitic treated patients and an animal model. For this, a line of work was followed comparing the immune response in cross-sectional studies. The patients with chronic Chagas disease (CCP) were classified, according to the American College of Cardiology/American Heart Association indications (**Table 1**), as: without cardiac damage (IND), and with cardiac damage (CCC), and their results compared with those of individual without *T. cruzi* infection (HD) and patients with non-Chagas heart disease (NCC) (**Figure 1**). The main findings and the perspectives of work in the current era of OMICS and possible scenarios when including other actors of the immune response are shown and discussed below.

**Table 1:** Chronic Chagas cardiomyopathy classification of the American College of Cardiology/American Heart Association.

Stage	ECG* findings	Heart size	LVEF**	NYHA*** functional class
A	Normal	Normal	Normal	I (no symptoms)
B	Abnormal	Normal	Normal	I (no symptoms)
C	Abnormal	Increased	Decreased	II (symptoms with ordinary activity) III (symptoms with less than ordinary activity)
D	Abnormal	Increased	Decreased	IV (symptoms at rest)

*ECG, electrocardiogram.

**LVEF, left ventricular ejection fraction.

***NYHA, New York Heart Association.

## 2 Search for immunogenic antigens

*T. cruzi* is a highly complex and variable parasite with substantial genomic plasticity, enabling it to evade the host immune response, thereby successfully establishing the infection and perpetuating itself by infecting a new host. The genetic variability of *T. cruzi* is not only evidenced by the existence of seven genotypes or discrete typing units (DTUs) (Zingales et al., 2012) from TcI to TcVI, and TcBat but also by the genetic variability within each DTU, as occurs for the DTU TcI (Talavera-López et al., 2021). Although the results of the first *T. cruzi* genome project were reported in 2005 (El-Sayed et al., 2005), given the repetitive nature of the parasite genome, it was not possible to reconstruct the complete chromosomes at that time. However, in 2018, 98.5% of the genome from the TcI Sylvio X10/1 strain was published (Talavera-López et al., 2018), and in 2021 (Talavera-López et al., 2021), the genomes of 33 isolates belonging to this same DTU were published. These studies found that the greatest source of diversity lies in the multigene families that code for surface proteins (trans-sialidases, mucins, and mucin-associated surface proteins (MASP)) involved in the invasion, pathogenesis, and evasion of the immune response. These genes are found in regions with a high concentration of retrotransposons and other repeated elements that give the parasite the micro-homologous sequences required for the occurrence of recombination events that lead to the generation of antigenic diversity in these proteins (Herreros-Cabello et al., 2020). In addition to the high number of parasite proteins (approximately 12,000 putative proteins) and their antigenic variability, the existence of sequence similarity between parasite and human proteins is added to this scenario (Girones et al., 2005). The lack of identification of immunodominant fragments from parasite proteins recognized by T cells in patients with Chagas disease has also been influenced by other factors, such as protein abundance; proteasome peptide generation and transport; peptide affinity for the major histocompatibility complex (MHC); composition of the TCR repertoire, and competition between T cells for antigen-presenting cells, among others. However, a number of CD8^+^ T cell parasitic-derived epitopes have been described (Ferragut et al., 2021).

Therefore, in the 2000s, after conducting a literature review and bioinformatic analyses with the information available at that time, it was opted to study the 11kDa membrane protein of kinetoplastids (KMP11). Notably, KMP11 has several characteristics that make it a very attractive target for study. It is exclusive to kinetoplastids (Cuervo et al., 2007), it has been reported in all parasite-DTU genomes published thus far (reviewed on TritrypDB in August 2022), and it is expressed in all *T. cruzi* stages (consulted on TritrypDB in July 2022) as well as in *Trypanosoma rangeli* (Diez et al., 2005; Diez et al., 2008). Interestingly, *T. rangeli* is a parasite that is not pathogenic for humans but is distributed like *T. cruzi* in the Andean countries of Latin America and has been used as a model for the induction of immunity against *T. cruzi* (Puerta, 2007).

Thus, in this search path, we first explored whether the KMP11 protein was capable of naturally inducing humoral or cellular immune responses. For this, the levels of IgG antibodies against the KMP11 protein were measured in CCP and HD. It was found that, unlike HD, CCP had IgG antibodies against the KMP11 protein, with IgG1 being the predominant subclass of IgG antibodies which are more prone to complement fixation (Flechas et al., 2009). Also, by flow cytometry experiments, it was observed that CD4^+^ T cells from CCP were able to respond to the stimulus of KMP11 secreting interferon-gamma (IFN-γ) but not interleukin 4 (IL-4). No response was observed in CD4^+^ T cells from NCC and HD (Cuellar et al., 2009). These findings showed that KMP11 triggers B cell, and CD4^+^ T cell responses in the natural context of the disease, with a Th1 profile response.

To study the response of CD8^+^ T cells, ELISPOT assays were initially carried out using the TcTLE peptide (originally named K1) corresponding to amino acids 4-12 (TLEEFSAKL) of the N-terminal region of the KMP11 protein. An HLA-humanized mouse model was used to show that TcTLE was a cytotoxic epitope restricted to HLA-A*0201 (Planelles et al., 2001). ELISPOT results indicated that the TcTLE peptide is recognized and presented by the CD8^+^ T cells in 2 of out 12 HLA-A*0201^+^ CCP studied, producing IFN-γ with a relative frequency of 110-230 per million CD8^+^ T cells. In contrast, HLA-A*0201^+^ HD did not respond to the TcTLE peptide (Diez et al., 2006). Responses to HLA-A*0201-restricted peptide from the influenza matrix protein (MP-FLU) were found in 6 out of 12 CCP and 4 out of 10 HD, with an average frequency of 175 and 111 cells per million CD8^+^ T cells, respectively. Moreover, a flow cytometric degranulation assay using CD107a/b (a marker of exocytic vesicles) showed that CCP responders had TcTLE-specific cytotoxic CD8^+^ T cells (Diez et al., 2006). These results indicated that TcTLE peptide is processed, presented, and recognized by CD8^+^ T cells in the natural context of the disease (**Table 2**).

To characterize the TcTLE-specific CD8^+^ T cells HLA-A2 soluble tetramers were used and the number of CCP to be studied was expanded. The tetramers were produced by the National Institute of Health (NIH) Tetramer Facility (Atlanta, GA, USA). It was found that 15 out of 19 patients had TcTLE-specific CD8^+^ T cells (0.09-0.34%) without differences according to disease stage or severity (Lasso et al., 2010). Of note, five of these responders had other HLA allele subtypes, including HLA-A*0205, A*0222, A*0226, A*0259, and A*0287. Thus, a phenotypic and functional comparison of TcTLE-specific CD8^+^ T cells from non-HLA-A*0201 and HLA-A*0201^+^ Chagas patients was performed. The results showed that HLA-A*0201^+^ and non-HLA-A*0201 CCP had a predominant effector memory CD8^+^ T cell phenotype (CCR7^-^ CD62L^-^). In addition, similar frequencies of TcTLE-specific CD8^+^ T cells between HLA-A*0201^+^ and non-HLA-A*0201 CCP producing IL-2, IFN-γ and perforin were observed (Lasso et al., 2010). These findings showed that the TcTLE peptide, which is highly recognized by CCP, is an immunodominant promiscuous epitope presented by HLA-A2 supertype molecules (**Table 2**).

During our studies, it was observed that the CD8^+^ T cells of some HLA-A2-negative patients were also able to recognize the TcTLE peptide. Therefore, using the same methodological approach, a group of 36 HLA-A2-negative CCP was analyzed, founding that 28 of them had CD8^+^ T cells capable of recognizing the peptide with frequencies between 0.07-0.37%. When typing the HLA-A alleles of these responding patients, it was determined that 7 of the 28 CCP had HLA-A homozygous alleles: A24 (5 patients), A23 (1 patient), and A01 (1 patient), which belong to the HLA-A24 and A1 supertypes (Lasso et al., 2016a). The remaining 21 patients had HLA-A heterozygous alleles, being the most prominent ones HLA-A24 and A68. The most common allele subtype was HLA-A*2402 (16 patients), which belongs to the A24 supertype, followed by HLA-A*6802 (6 patients) belonging to the A2 supertype. Additionally, the HLA-A*3002/A*3201 alleles from the HLA-A1 supertype were detected in one patient. All patients presented CD8^+^ T cells producing at least one cytokine after TcTLE-peptide stimulation (Lasso et al., 2016a). These data reinforced that TcTLE is a promiscuous epitope presented not only by the HLA-A2 supertype alleles but also by the HLA-A24 and A1 supertypes (**Table 2**).

**Table 2:** Features of specific TcTLE-CD8^+^ T cells from patients with Chronic Chagas Disease (CCP).

Assay	CCP responders CD8+ T cell frequency range	CCP H LA/Supertype	Phenotype	Cytokine production	Cytotoxic activity	References
ELISPOT	2/12 (110 - 230 x 10^6)	HLA-A*0201/A2	ND	IFN-γ	ND	Diez et al., 2005
Degranulation by FC	2/2	HLA-A*0201/A2	ND	ND	CD107a/b	Diez et al., 2005
Tetramers	15/19 (0.07-0.34%)	HLA-A*0201/A2	Predominant CCR7^-^ CD62L^-^	IFN-γ IL-2	Perforin	Lasso et al., 2010
		HLA-A*0205/A2				
		HLA-A*0201/A2				
		HLA-A*0222/A2				
		HLA-A*0226/A2				
		HLA-A*0229/A2				
		HLA-A*0286/A2				
	28/36 (0.07-0.37%)	HLA-A*0101/A1	ND	IFN-γ IL-2 TNF-α	Perforin CD107a/b	Lasso et al., 2016a
		HLA-A*0301/A3				
		HLA-A*1101/A3				
		HLA-A*2301/A24				
		HLA-A*2402/A24				
		HLA-A*2403/A24				
		HLA-A*2404/Unclassified				
		HLA-A*2414/A24				
		HLA-A*2902/A1-A24				
		HLA-A*2903/A1-A24				
		HLA-A*2904/Unclassified				
		HLA-A*3001/A1-A3				
		HLA-A*3002/A1				
		HLA-A*3004/A1				
		HLA-A*3010/Unclassified				
		HLA-A*3101/A3				
		HLA-A*3109/A3				
		HLA-A*3201/A1				
		HLA-A*3301/A3				
		HLA-A*6801/A3				
		HLA-A*6802/A2				

A frequent problem in designing multiepitope vaccines is the MHC restriction of the T cell responses, and the enormous diversity of HLA alleles in a population. Therefore, the identification of promiscuous epitopes such as TcTLE, which are presented in the context of different HLA alleles and recognized by the T cells of a large percentage of the population, constitutes a very striking target to develop immune intervention measures against infection. Importantly, it is estimated that more than 95% of individuals carry at least one MHC allele that is classified into one of the 6 most common HLA class I supertypes, and it is considered that at least 50% of individuals express alleles belonging to the HLA-A2 supertype (Reche and Reinherz, 2005). Similarly, in the population of Latin America where Chagas disease is endemic, the most common HLA-A alleles are: HLA-A2 (28%), HLA-A24 (11%), and HLA-A68 (5%) in Brazil (Bardi et al., 2012; Bortolotto et al., 2012); HLA-A2 (50%), HLA-A24 (14%), and HLA-A68 (10%) in Bolivia (Martinez-Laso et al., 2006); HLA-A2 (63.4%), HLA-A30 (10.2%), HLA-A24 (6.6%), and HLA-A68 (5.4%) in Peru (Moscoso et al., 2006); and HLA-A2 (25.5%), HLA-A24 (23%), and HLA-A68 (6.0%) in Colombia (Arias-Murillo et al., 2010; Arrunategui et al., 2013). In particular, these reports indicate that more than 45% of the Latin American population has HLA-A2, A24, or A68 alleles, capable of presenting the TcTLE peptide and inducing a CD8^+^ T cell response through effector functions such as cytokine secretion and cytotoxic activity.

Importantly, the multifunctional response induced by the TcTLE peptide was also determined by the simultaneous detection of cytokines production and the cytotoxic capacity in antigen-specific CD8^+^ T cells (**Table 2**). In models of chronic infectious diseases, profiles of multifunctional and monofunctional T cells have been described. In the first profile, a single cell is capable of producing several cytokines or molecules with cytotoxic function simultaneously, and in the second case, a single cell produces a one cytokine or a molecule with cytotoxic function. Cellular multifunctionality has been associated with protection in several infectious models (Darrah et al., 2007). After TcTLE peptide stimulation, and measuring of five effector functions (IFN-γ, TNF-α, IL-2, perforin, and CD107a/b), it was found that the peptide-specific CD8^+^ T cells from IND and CCC patients produced four, three, and two of these functions. Generally, IND patients had a higher frequency of CD8^+^ T cells exhibiting four or two functions than those with cardiac manifestations. The most predominant profile, including four positive effector functions, was CD107a/b, IFN-γ, perforin, and TNF-α (Lasso et al., 2016a). Collectively, all these findings indicate that the HLA class I restricted-TcTLE peptide is also capable of inducing a multifunctional effector response. In chronic viral infections, the temporary loss of cytokines begins with IL-2, an important factor in the activation and differentiation of T cells, continuing with the loss of TNF-α secretion and finally IFN-γ (Wherry, 2011). However, in the case of chronic Chagas disease, the secretion of TNF-α is usually the last cytokine to be lost (Lasso et al., 2015).

To enhance the immunogenic properties of TcTLE, three substitutions were made in secondary anchor residues at positions 3, 6, and 7 of the TcTLE peptide (E3L, S6V, and A7F). In contrast to bioinformatics predictions, the TcTLE-modified peptide was found to have lower binding affinity and stability than the original peptide. Nevertheless, CD8^+^ T cells from CCP recognized the TcTLE-modified peptide producing TNF-α and IFN-γ along with the expression of CD107a/b, although to a lesser extent than the response triggered by the original TcTLE peptide (Lasso et al., 2016b). Overall, although the amino acids at positions 3, 6, and 7 of TcTLE are critical for secondary peptide affinity to HLA, it seems that they have a limited effect on the immunogenic properties of the TcTLE epitope.

**Figure 2:**
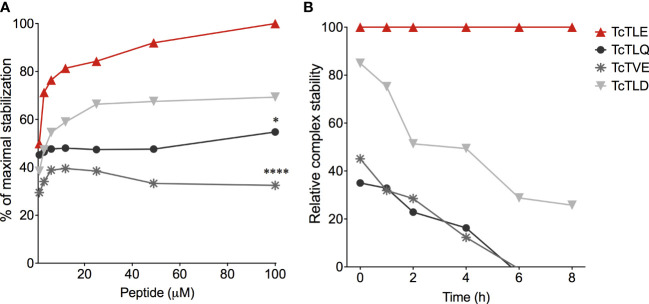
Binding assays of TcTLE homologous peptides to HLA-A*0201. **(A)** Binding affinity assay of peptides homologs to the HLA-A*0201 molecule using the original TcTLE peptide as an internal control. The binding affinity of each sequence was defined with different peptide concentrations. **(B)** Binding stability assay of the peptide/HLA-A*0201 complex normalized to the relative stability of the complex with the TcTLE peptide. The binding stability of each peptide to HLA-A*0201 was analysed at different times using 100 mM of each peptide. The two tests were performed in duplicate. Each point represents the median of 4 experiments performed independently. The *p* values were calculated using Mann-Whitney *U* test. *p < 0.05, ****p < 0.0001.

On the other hand, given the concept of altered peptide ligands (APLs), defined as homologs sequences that present substitutions of a few amino concerning the original peptide that can affect its performance (Unanue, 2011), in 2009 we explored whether the parasite could express peptides similar to TcTLE. Thus, Blast/p analysis using the *T. cruzi* GeneDB (2009) database yielded several sequences which were studied to define their ability to bind to HLA-A*0201. For this, at that time, SYFPEITHI (http://www.syfpeithi.de) (Rammensee et al., 1999), NetMHC 3.4 (https://services.healthtech.dtu.dk/service.php?NetMHC-3.4) (Nielsen et al., 2003), and HLA Peptide Binding Predictions (http://www-bimas.cit.nih.gov/molbio/hla_bind) (Parker et al., 1994) tools were used, followed by *in silico* prediction of the proteasome peptides processing (MHC-I Antigenic Peptide Processing Prediction: http://www.mpiib-berlin.mpg.de/MAPPP). Thereby, we obtained three peptide sequences homologs to TcTLE (called TcTLD, TcTLQ, and TcTVE) which were tested *in vitro* to corroborate their binding capacity to HLA-A*0201 and the stability of the resulting HLA/peptide complex (**Table 3**). All peptides bound with different affinities to HLA-A*0201 molecules. Peptide TcTLE showed a higher affinity, followed by peptide TcTLD, TcTLQ, and TcTVE (**Figure 2A**). Taking the TcTLE/HLA-A*0201 complex as a reference, the complex formed by TcTLD was reduced by approximately 50-60% after 8 hours of the initial incubation, while the complex formed with the other two homologs remained stable for only 6 hours (**Figure 2B**). It was shown that selected peptides bind to HLA with different affinities to that of the original peptide.

**Table 3:** Bioinformatic predictions of HLA-A*0201 binding and proteasome processing of peptides homologous to TcTLE.

Peptide	Protein (aminoacid position)	Access number	Sequence	*Score*			Cleavage prediction by the proteasome
				SYFPEITHI^a^	NetMHC 3.4^b^	BIMAS^c^	
TcTLE	KMP-11 protein of *T. cruzi* (4 - 11)	AAF04809	TLEEFSAKL	26	0.51	21.3	0.99
TcTLQ	CEP-19 like protein (338 - 346)	TcCLB.503583.50	TL**Q**EF**K**AK**M**	21	0.35	77.4	0.57
TcTVE	Hypothetical protein of *T. cruzi* (107 - 115)	TcCLB.505009.49	T**V**EE**S**S**ER**L	15	0.11	0.24	0.52
TcTLD	Hypothetical protein of *T. cruzi* (27 - 35)	TcCLB442.629.9	TL**D**E**VA**AK**M**	20	0.46	8.1	0.50

The amino acids that vary with respect to the original TcTLE peptide are shown in bold. **a:** It is based on the preference of amino acids in each position with respect to the HLA. The maximum score for binding to HLA-A*0201 is 36. **b:** A high score is associated with a high binding affinity. **c:** Predicts the mean dissociation time of the peptide/HLA complex. In predicting cleavage by the proteasome, peptides with values closer to 1 are more likely to be cleaved by the proteasome.

**Figure 3:**
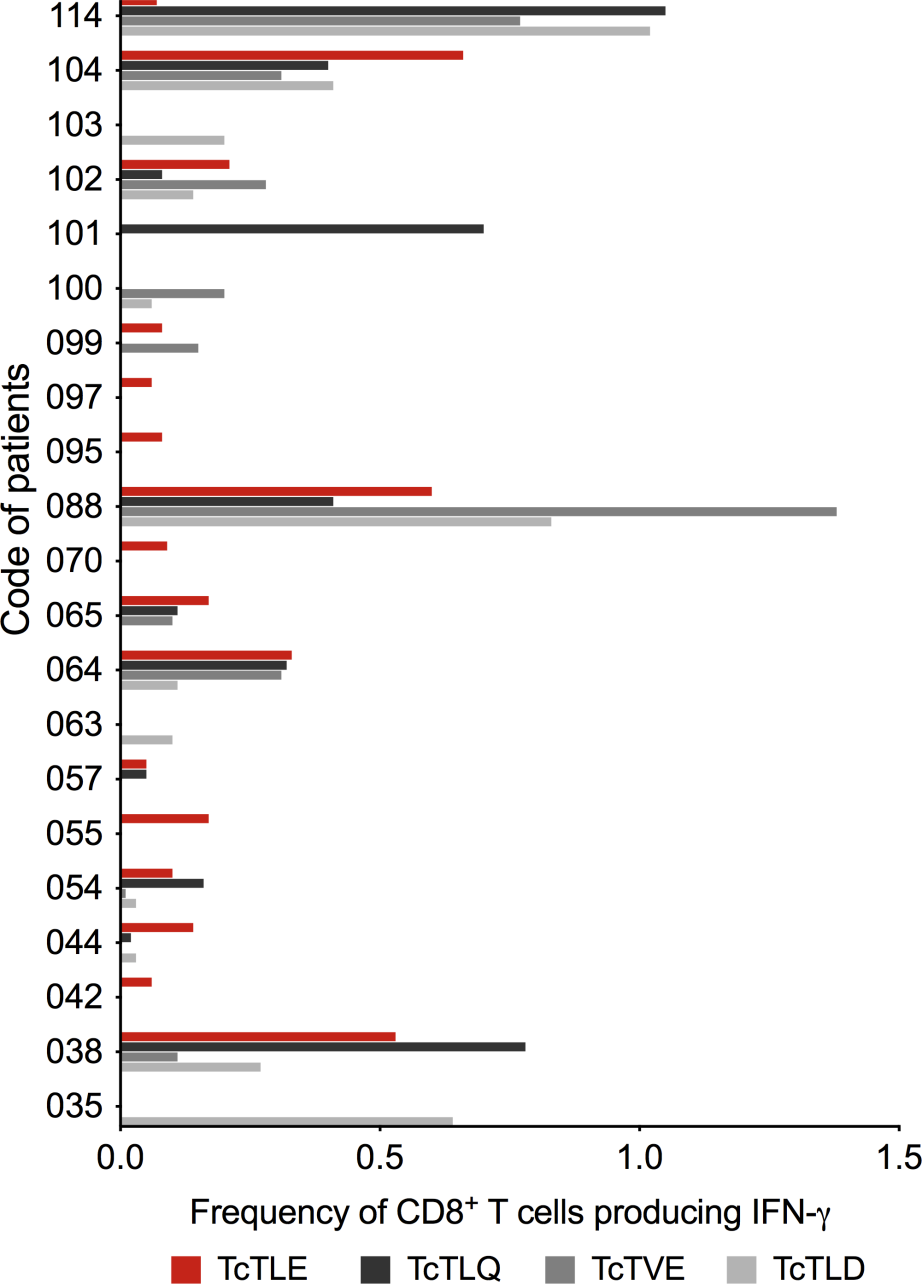
Frequency of CD8^+^ T cells that produce IFN-γ after stimulation with the original TcTLE peptide and homologous peptides after 6 hours of culture with each peptide in CCP (n = 21). The cut-off point for considering a positive response (> 0.05%) was subtracted from each peptide.

**Figure 4:**
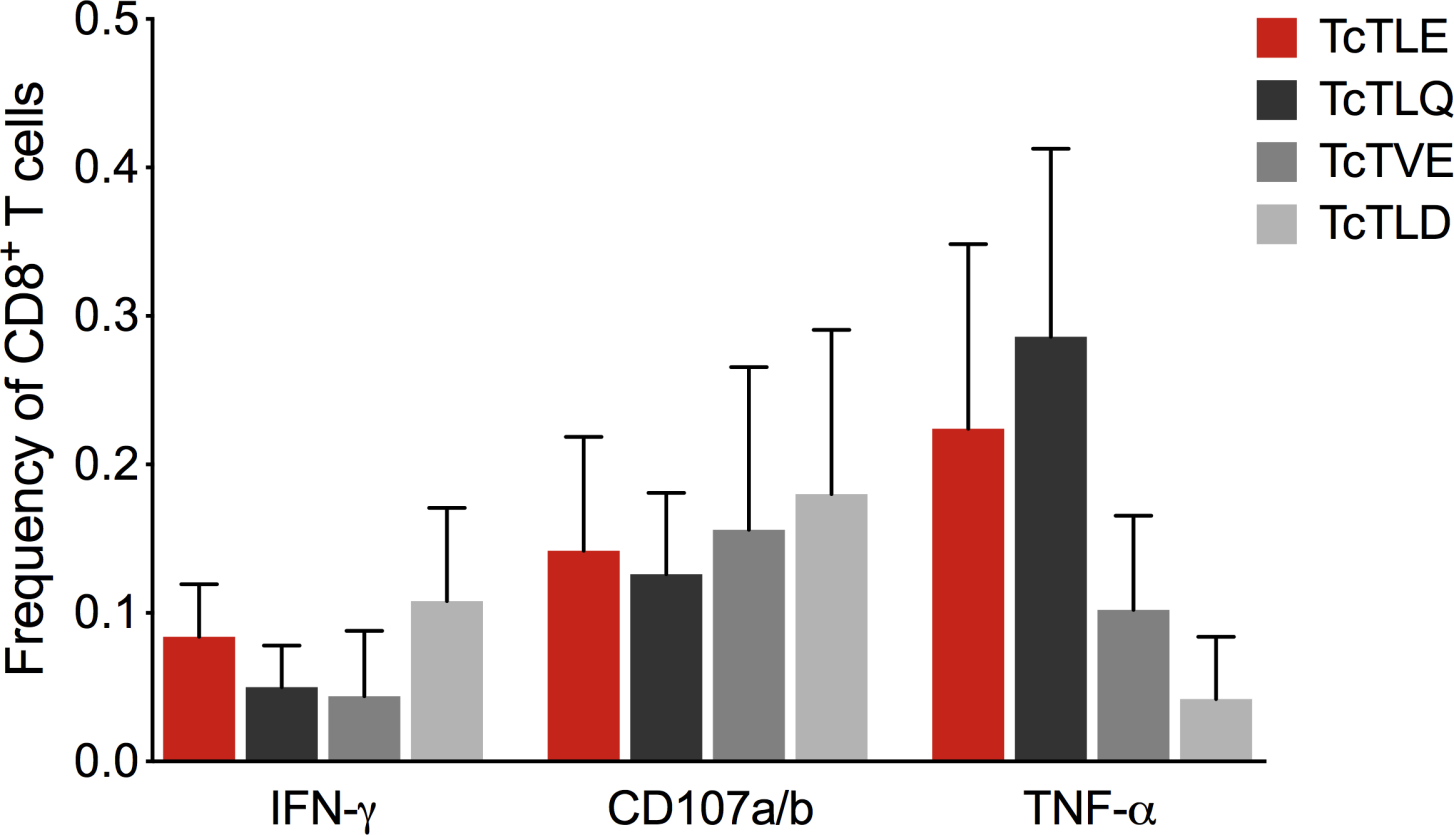
Frequency of CD8^+^ T cells in CCP (n = 5) that secrete cytokines or express CD107a/b after 6 hours of culture with the original TcTLE peptide and homologous peptides. Each column shows the median and frequency range of CD8^+^ T cells expressing each molecule tested.

Since the TritrypDB-annotated transcriptomic and proteomic analyses of these sequences indicate their transcription and expression in the parasite, it was examined whether the TcTLE homologs peptides are processed, presented and recognized by CD8^+^ T cells during natural infection. For this, the frequency of specific CD8^+^ T cells from CCP that produce IFN-γ was evaluated after stimulation with each peptide. It was found that the three homologous peptides are capable of inducing a response in CD8^+^ T cells through the production of IFN-γ with highly variable profiles and, in some cases, with higher frequencies than those obtained with the TcTLE peptide. Indeed, 16 of 21 (76%) CCP had CD8^+^ T cells that recognized TcTLE peptide, 11 of 21 (52%) for TcTLQ peptide, 10 of 21 (48%) for TcTVE peptide, and 12 of 21 (57%) recognized TcTLD peptide (**Figure 3**). Interestingly, 5 CCP had CD8^+^ T cells that recognized only TcTLE peptide, 1 CCP had CD8^+^ T cells that recognized only TcTLQ peptide, and 3 CCP had CD8^+^ T cells that recognized only TcTLD peptide (**Figure 3**). These results suggest that, despite the homology between the peptide sequences, the cellular response to each peptide is independent. Knowing that homologous peptides are recognized by CD8^+^ T cells of CCP and induce the IFN-γ production, it was determined whether these sequences could also induce TNF-α production or cytotoxicity (CD107a/b) in CD8^+^ T cells of HLA-A2^+^ CCP. All peptides induced the production of IFN-γ and TNF-α and the degranulation of CD8^+^ T cells without significant differences between the three homologous sequences (**Figure 4**). These results show that TcTLE peptide homologs peptides are processed, presented and recognized by CD8^+^ T cells of CCP during the natural course of infection.

Later, to test the possible agonist or antagonist effect of the homologous sequences on the TcTLE peptide-specific CD8^+^ T cells response, it was determined whether the CD8^+^ T cells that respond to the stimulus with each homologous peptides are the same CD8^+^ T cells specific for TcTLE. Therefore, peripheral blood mononuclear cells (PBMCs) of 5 CCP were incubated with each peptide and subsequently labelled with the TcTLE/HLA-A02^+^ PE tetramer and anti-cytokine antibodies. Thus, the production of cytokines induced by each peptide on TcTLE-specific CD8^+^ cells population was determined by staining with soluble tetramers. It was found that TcTLE-specific CD8^+^ T cells respond only to TcTLE-stimulation, thereby producing TNF-α, IL-2, IFN-γ, or granzyme B (**Table 4**). In all, these results indicate that homologs sequences are not recognized by TcTLE-specific CD8^+^ T cells showing that these sequences do not act as TcTLE APLs. On the contrary, this study revealed three parasite new epitopes capable of inducing a functional CD8^+^ T cell responses. Thus, these three sequences derived from the putative protein of the centrosome of 19 kDa and two hypothetical parasite proteins (**Table 3**) provide new candidates for the rational design of immune therapies or vaccines against Chagas disease.

**Table 4:** Frequency of TcTLE-specific CD8^+^ T cells that respond to stimulation with homologous peptides.

Patient code	Antigen	Frequency of CD8^+^ T cells^a^			
		TNF-α^+^	IL-2^+^	IFN-γ^+^	Granzyme B^+^
QX-035	TcTLE	2.56	0.91	3.18	1.50
	TcTLQ	0.00	0.00	0.00	0.30 ^b^
	TcTVE	0.00	0.00	0.00	0.20 ^b^
	TcTLD	0.00	0.00	0.18 ^b^	0.00
QX-042	TcTLE	2.56	2.95	1.86	1.30
	TcTLQ	0.00	0.00	0.04	0.00
	TcTVE	0.00	0.00	0.00	0.00
	TcTLD	0.00	0.00	0.00	0.00
QX-102	TcTLE	0.67	1.30	0.25 ^b^	3.90
	TcTLQ	0.00	0.00	0.00	0.00
	TcTVE	0.00	0.00	0.00	0.00
	TcTLD	0.00	0.00	0.00	0.00
QX-103	TcTLE	0.39 ^b^	1.66	1.63	5.30
	TcTLQ	0.00	0.00	0.00	0.00
	TcTVE	0.00	0.00	0.00	0.00
	TcTLD	0.00	0.00	0.00	0.00
QX-104	TcTLE	3.42	1.00	0.56	3.80
	TcTLQ	0.17 ^b^	0.05 ^b^	0.00	0.00
	TcTVE	0.14 ^b^	0.04	0.00	0.00
	TcTLD	0.02	0.00	0.03	0.00

a The positive response of cytokine production or cytotoxic activity (bold) was defined as > 0.05% after subtraction of background noise (culture without antigen). However, the frequency of CD8+ T cells cytosine producers must have at least 10 events to be considered positive.

b Frequencies > 0.05% that have fewer than 10 events to be considered positive.

The QX-035, QX-042, QX-102 and QX-104 patients were classified as asymptomatic and QX-103 as symptomatic.

Because of the promising TcTLE peptide findings, we decided to characterize its secondary structure and explore the humoral immune response against this peptide. Circular dichroism and NMR spectroscopy analysis revealed that in solution TcTLE peptide adopts an α-helical conformation (Díez et al., 2007). Remarkably, adopting a defined secondary conformation seems to be a characteristic of antigenic peptides (Dyson and Wright, 1995). ELISA tests showed that 56% of CCP had anti-TcTLE and 86% anti-KMP11 antibodies. Similarly, in the IND group, 28% presented anti-TcTLE antibodies and 68% anti-KMP11 antibodies. In contrast, no reactivity was observed in sera from HD and patients with another infectious disease (tuberculosis) (Díez et al., 2007). Likewise, for KMP11, the antibody subclasses specificity to the TcTLE peptide were IgG1 and IgG3 (Díez et al., 2007; Flechas et al., 2009). Overall, these results support the idea that the TcTLE peptide also acts as a B cell-epitope during Chagas disease. In addition, since the KMP11 protein is part of the cytoskeleton of the parasite (Thomas et al., 2000), it was studied whether antibodies against the TcTLE peptide can alter the motility of the parasite and inhibit its invasion to the host cell. Rabbit sera raised against the TcTLE peptide showed recognition of the KMP11 recombinant protein by ELISA and Western blot as well as of the native protein in include amastigotes evaluated by immunofluorescence and flow cytometry assays (Diaz-Soto et al., 2012; Finkelsztein et al., 2015). Trypomastigotes exposed to post-immunization TcTLE sera had qualitative alterations in motility and significantly slower flagella speed (45.5 μm/s) than those exposed to pre-immunization sera (69.2 μm/s). Invasion assays, previously standardized by our group (Vargas-Zambrano et al., 2013; Duran-Rehbein et al., 2014), showed a significant reduction in the trypomastigote infection rate of fibroblast and glioblastoma cell lines when parasites were pre-incubated with anti-TcTLE rabbit serum (Diaz-Soto et al., 2012; Finkelsztein et al., 2015). Accordingly, computational modelling predicted that the TcTLE sequence conserved its α-helical configuration into the protein, and some of the amino acid residues appear accessible for recognition by antibodies *in vivo* (Diaz-Soto et al., 2012). Collectively, these data support the idea that the TcTLE peptide induces antibodies that can have a potential role in protective immunity in Chagas disease.

In summary, (i) TcTLE, an α-helical peptide exclusive of kinetoplastids, is conserved among the different *T. cruzi* strains and DTUs, and between *T. rangeli* KP1^+^ and KP1^-^ strains, which added to the abundancy of the KMP11 protein in all parasite stages, increases the TcTLE available amount for immune recognition. (ii) TcTLE peptide is presented, processed, and recognized during the natural history of the disease in the context of different HLA-A molecules, which enables it as a promiscuous epitope capable of inducing immune response in a large number of individuals. (iii) TcTLE peptide elicits a multifunctional cellular immune response characterized by cytokine production and cytotoxic activity, which has been associated with protection. (iv) TcTLE peptide triggers a humoral response capable of decreasing the parasite movement and infective capacity. Therefore, this T and B cells-epitope could be a useful antigen for monitoring of the specific immune response and developing of biomarkers, and vaccines or immunotherapy strategies. Interestingly, *in silico* studies to discover T and B epitopes of the parasite identified a B epitope of the KMP11 protein but failed to identify the TcTLE peptide (Michel-Todó et al., 2019).

## 3 Deciphering CD8^+^ T cells in Chagas disease

The total CD8^+^ T cell pool in CCP was characterized trough the analysis of its proliferative capacity, expression of costimulatory and activation-signals transducer molecules, the ability to respond to antigens of another microorganism, the activation state, presence of CD4^+^/CD8^+^ double-positive T cells, phenotyping of the different subpopulations of memory T cells, and expression of inhibitory receptors by using flow cytometry techniques previously standardized by us (Mateus et al., 2013; Lasso et al., 2019).

The proliferative capacity of T cells is essential to mount a protective memory immune response (Harty and Badovinac, 2008). After conducting several studies, our group showed that, unlike CD8^+^ T cells of HD, CD8^+^ T cells of IND and CCC present alterations in proliferation while expressing a lower percentage of costimulatory and activation-signals transducer molecules (Giraldo et al., 2013; Gómez-Olarte et al., 2019). Likewise, analysis of infiltrating CD8^+^ T cells in two cardiac explants showed similar results (Giraldo et al., 2013). Notably, it was observed that if mitogen-stimulated CD8^+^ cells of HD are exposed to parasitic antigens, they also decrease their proliferative capacity and their expression of CD28 and CD3ζ (Giraldo et al., 2013; Gómez-Olarte et al., 2019). In other words, the decrease in the proliferative capacity of CD8^+^ T cells in patients is conditioned by the presence of the parasite and can be partially explained by the decrease in costimulatory and activation-signal transducer molecules. Similar findings have been reported in cytomegalovirus infection in which a normal response to MP-FLU peptide from Influenza virus, and a decreased proliferative capacity of specific T lymphocytes were observed (Lu et al., 2022a).

It was also studied whether the alteration in the functionality of CD8^+^ T cells was nonspecific or specific to parasite antigens; we studied the CD8^+^ T cell response of CCP against an antigen of another microorganism. In this case, the MP-FLU peptide (GILGFVFTL) derived from the influenza virus, which is also restricted to HLA-A2 (Lasso et al., 2012). The results showed that MP-FLU peptide-specific CD8^+^ T cells presented similar frequencies in the five HLA-A2^+^ HD (0.12-0.29%, mean 0.17%, SD = 0.07) and the 13 HLA-A2^+^ CCP (0.12-0.37%, mean 0.21. %, SD = 0.07). In addition, MP-FLU-specific CD8^+^ T cells were predominantly effector memory T cells (T_EM_: CCR7^-^CD62L^-^), distributed in the early or intermediate/late differentiation stages, producing IL-2, IFN-γ, CD107a/b, and perforin without showing significant differences when compared with those from HD (Lasso et al., 2012). In all, these data support the hypothesis that parasite antigens induce a specific CD8^+^ T cell dysfunctionality in terms of reduced proliferation capacity and a prolonged activation state.

In several models of chronic infections, mainly viral infections, it has been shown that due to the constant antigenic stimulus of the infectious agent, T cells can remain in a state of continuous activation that decreases their functional capacity (Wherry, 2011). Thus, our studies revealed that CCP are characterized by a higher percentage of activated CD8^+^ T cells that co-express HLA-DR and CD38 molecules. Consistent with the persistence of the parasite in the chronic phase of the disease, this phenomenon of prolonged state of activation may be induced by parasitic antigens. Interestingly, in a study to determine whether the CCP had doubly positive (DP) T cells, that is, whether that they express both CD4 and CD8 (CD4^high^CD8^low^ or CD4^low^CD8^high^), it was found that not only patients express a higher percentage of DP T cells than HD but also that DP T cells of the patients had a higher expression of HLA-DR and CD38 (Giraldo et al., 2011). In agreement, the increased presence of activated DP cells associated with severe cardiac forms of human chronic Chagas disease was also described (Morrot et al., 2011). Moreover, our immunochemistry analysis revealed that DP T cells also infiltrate the cardiac tissue of CCP, potentially increasing the population of activated T cells (Giraldo et al., 2011). Nonetheless, recently a protective role of double-positive cells has been suggested in viral models such as Hantaan virus infection (Zhang et al., 2022).

Memory CD8^+^ T cells can be categorized based on their distinct differentiation stages and functional activities, as proposed by Mahnke et al., 2013: stem cell memory (T_SCM_), central memory (T_CM_), transitional memory (T_TM_), effector memory (T_EM_), and terminal effector (T_TE_) cells (**Table 5**) (Mahnke et al., 2013). Changes in the distribution of CD8^+^ T cell subsets could highlight the behaviour of cellular immunity during the natural history of infections. To characterize these total and specific CD8^+^ T cell memory subsets, we compared them in CCP with different degrees of cardiac dysfunction. We observed a decreased frequency of total T_SCM_ along with an increased frequency of T_TE_ in CCP with severe disease. These changes observed for the T_SCM_ and T_TE_ subsets indicated a negative correlation in both the frequency and the absolute numbers of CD8^+^ T cells in all CCP analyses. Of note, antigen-specific T_SCM_ cells measured by cytokine-producing cells were not detectable in CCP with severe forms of the disease, suggesting a possible protective role of the T_SCM_ in the outcome disease as these cells may be involved in repopulating the T cell pool that controls infection (Mateus et al., 2015). Remarkably, T_SCM_ cells have been associated with protection in other models such as the Hepatitis C model (Lu et al., 2022b).

**Table 5:** Phenotypes of memory T cell subsets in humans and mice.

T cell subsets	Humans	Mice
Memory stem T cells (T_SCM_)	CD45RA^+^CCR7^+^CD27^+^CD28^+^CD127^+^CD95^+^	CD44^-^CD62L^+^CD122^+^
Central memory T cells (T_CM_)	CD45RA^-^CCR7^+^CD27^+^CD28^+^CD127^+^CD95^+^	CD44^+^CD62L^+^
Effector memory T cells (T_EM_)	CD45RA^-^CCR7^-^CD27^+^CD28^+^CD127^+/-^CD95^+^	CD44^+^CD62L^-^
Terminal effector T cells (T_TE_)	CD45RA^+-^CCR7^-^CD27^-^CD28^-^CD127^-^CD95^+^	Undetermined

The expression of inhibitory receptors in T cells was initially associated with preventing immune hyperactivation and autoimmunity. However, in the past decade, numerous inhibitory receptors have been identified as regulators of both the function and proliferation of T cells. Indeed, their expression and co-expression under conditions of antigenic persistence or chronic infection have been associated with T cell exhaustion (Nakamoto et al., 2009; Wherry, 2011). In **Table 6**, some of the inhibitory molecules related to the depletion state of T cells are depicted. To elucidate the functional state of CD8^+^ T cells in CCP, the presence of these receptors in the CD8^+^ T cells, were assessed. It was observed that, compared with the HD, the CCP displayed a higher frequency of total CD8^+^ T cells that expressed the inhibitory receptors 2B4, CD160, PD-1, TIM-3, and CTLA-4 and co-expressed PD-1 and CTLA-4 on the one hand, and 2B4, CD160, and TIM-3 on the other (Lasso et al., 2015). When comparing the expression and co-expression of these receptors between IND, and CCC, it was found that the expression of CD160, PD-1, and CTLA-4 was significantly increased in CCC, as was the co-expression of PD-1, and CTLA-4 on one side, and 2B4, CD160, and TIM-3 on the other (Lasso et al., 2015).

**Table 6:** Inhibitory receptors described as regulators of T cell activation.

	PD-1 (C D279)	2B4 (C D244)	CTLA-4 (CD152)	CD160	TIM-3 (C D366)
*Functions*	Immune homeostasis Central and peripheral tolerance Inhibits proliferation and decreases effector functions	T cell and NK activation or inhibition receptor	Immune homeostasis Central and peripheral tolerance	T cell activation or inhibition receptor Cell proliferation modulation	Cell proliferation and effector functions of T cells modulation induces apoptosis Increases Treg-mediated suppression
*Distribution*	T cells, B cells, NK cells, monocytes, macrophages, dendritic cells	NK, T cells, monocytes, basophils, eosinophils	T cells, thymocytes, B cells, monocytes, granulocytes, CD34+ stem cells, fibroblasts, embryonic cells	NK cells, T cells	T cells, B cells, NK cells, Antigen-presenting cells (APCs)
*Ligand*	PD-L1 (CD274) PD-L2 (CD273)	CD48	CD80 (B7-1) CD86 (B7-2)	HVEM, CMH-I	Galectin-9, CEACAM-1
*Distribution of ligands*	(PD-L1) T cells, B cells, APCs, cells of mesenchymal origen, thymocytes *(PD-L2)* B cells, APCs, mast cells	Cells of hematopoietic origin	T cells, APCs	T cells, B cells, DCs, macrophages	T cells, eosinophils, endothelial cells, APCs
*Inhibitory mechanism*	Immunoreceptor tyrosine-based inhibitory motif (ITIM)/immunoreceptor tyrosine-based switch motif (ITSM)	ITIM/ITSM	Competition between costimulatory and coinhibitory signal molecules	Competition between costimulatory and coinhibitory signal molecules	Mediated by tyrosine motifs

Thus far, it is clear that CD8^+^ T cells population of CCP presents a dysfunctional state characterized by a prolonged hyper-activation state along with an increase of inhibitory receptors (2B4, CD160, PD-1, TIM-3, and CTLA-4) expression, an increase of specific terminal effector T cells (T_TE_), a decrease of proliferative capacity, a decrease of stem cell memory (T_SCM_) frequency, and a decrease of CD28 and CD3ζ as the severity of the disease progresses.

One of the functional markers of lymphocytes is their ability to secrete cytokines or produce molecules with cytotoxic capacity after antigenic stimulation. Thus, the functional response of parasite-specific CD8^+^ T cells, specifically against whole parasite antigens from the trypomastigote stage and KMP11, were determined. A functional profile of specific-CD8^+^ T cells among 24 CCP revealed a higher frequency of monofunctional CD8^+^ T cells in the most severe patients (CCC) with IFN-γ or TNF-α-producing cells (Mateus et al., 2015).

Similar to other chronic diseases, the progressive loss of certain functional activities during *T. cruzi* infection might result in the inability to control the parasite. To examine this hypothesis, we evaluated the differentiation and cell effector function of CD8^+^ T cells and characterized the expression of inhibitory receptors and the presence of the parasite in the peripheral blood of Chagas patients. The results showed that CCC had a higher frequency of T_TE_ than IND patients. A monofunctional CD8^+^ T cell response was observed in patients at an advanced stage, whereas patients at a less severe disease stage had *T. cruzi*-specific CD8^+^ T cells with three and four functions in response to parasite antigens. The frequency of CD8^+^ T cells producing granzyme B and perforin and those expressing inhibitory receptors was higher in CCC than in IND patients. It is worth noticing that the response to the KMP-11 protein had a behaviour very similar to that triggered by the parasite trypomastigote forms (Lasso et al., 2015).

**Figure 5:**
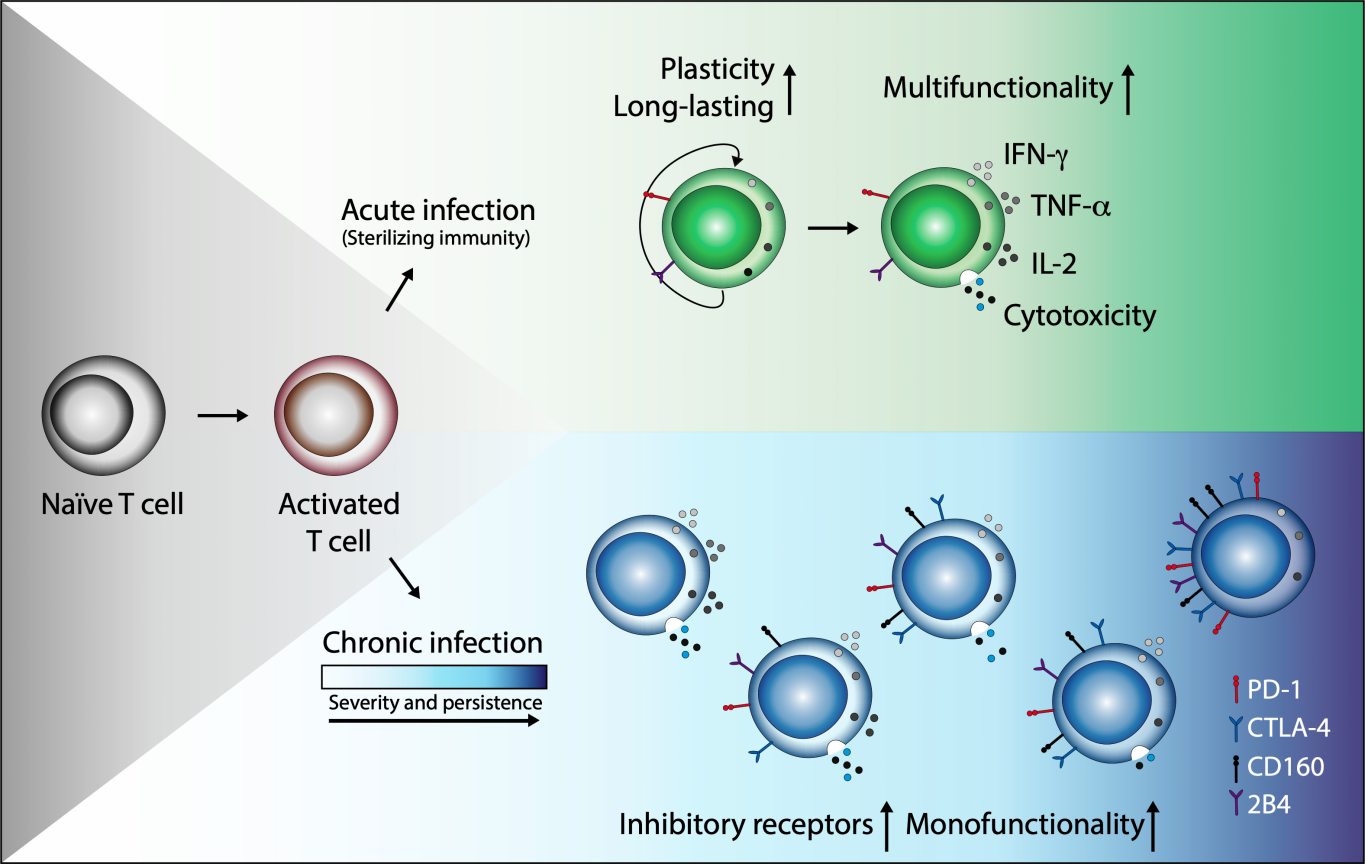
Chronic *T. cruzi* infection leads to clonal exhaustion characterized by monofunctional CD8^+^ T cell responses and coexpression of inhibitory receptors. In acute infection or after vaccination, antigen-presenting cells (APCs) capture and process antigens to activate naive T cells (T_N_). This activation process triggers a specific response to control the infection and eliminate the antigen (sterilizing immunity). After antigen clearance and resolution of inflammation, most T effector (T_Eff_) cells die; however, a small cell group persists and differentiates into memory T cells (T_Mem_) with remarkable proliferative potential, antigen-independent survival, and the ability to reactivate its effector functions in new encounters with the antigen. In contrast, in an infection where antigenic stimulation persists, although effector and memory T cell subsets are generated, T cells lose effector functions as the infection becomes chronic and increase the expression and co-expression of inhibitory receptors such as PD-1, CTLA-4, CD160, and 2B4. Some functions as cell proliferation or IL-2 production are initially lost. IFN-γ production can be subsequently reduced and TNF-α can decrease or be present as in the case of Chagas disease, and in severe states of exhaustion, cells may die by apoptosis.

In conclusion, in relation to IND patients, CCC had a lower number of parasite-specific CD8^+^ T_CM_ cells and a greater number of antigen-specific CD8^+^ T cells with late differentiation. Similarly, it was found that as the severity of the disease progresses, the frequency of multifunctional memory CD8^+^ T cells decreases, characterizing the most advanced phase of the disease by the predominance of monofunctional cells. It was also determined that the expression of inhibitory receptors is greater in the cells of CCC patients than in those of IND. Together, these findings suggest that during the course of Chagas disease, CD8^+^ T cells undergo clonal exhaustion characterized by a gradual loss of function characterized by impaired cytokine production, the presence of advanced differentiation, and increased inhibitory receptor co-expression (**Figure 5**).

Additionally, as TNF-α is usually the last cytokine to be lost during the course of chronic Chagas disease (Lasso et al., 2015), we analyzed if TNF-α is useful for assessing the pool of *T. cruzi*-specific T cells. For this, the expression of membrane TNF-α was determined in a total of 21 CCP, 11 HD, and 5 NCC. It was observed that CD8^+^ T cells from CCP, after *T. cruzi* antigen stimulation, displayed higher percentages of membrane-bound TNF-α than HD and NCC controls. Indeed, CCC were differentiated from IND based on the expression of membrane TNF-α, and in CD8^+^ T cells correlates with the degranulation (Ripoll et al., 2018). However, a greater number of patients are required to determine the use of this cytokine as a possible biomarker of disease progression.

## 4 CD8^+^ T cell quality: A biomarker of disease outcome?

The type of T cell immune response induced after infection or vaccination might be used as a possible predictor of protection against pathogens. Although T cells are heterogeneous, their immune quality is defined as the efficient maintenance of memory T phenotypes and a competent or multifunctional antigen-specific T cell response (Darrah et al., 2007; Ferreira et al., 2019). Efficient maintenance of early memory T cell subsets has been associated with the protection induced by successful vaccination and infection models (Ribeiro et al., 2014). In this regard, we established two approaches to explore this hypothesis.

The first approach was to compare the effect of the anti-parasitic treatment on the CD8^+^ T cell responses in IND compared with individuals without treatment. Therefore, their CD8^+^ T cells expression of inhibitory receptors and functionality were determined. The results showed that the anti-parasitic treatment in IND increased the frequency of T_CM_ and T_TE_ cells, decreased the co-expression of inhibitory receptors, and improved the antigen-specific CD8^+^ T cell response exhibited by the individual production of IFN-γ or IL-2 and a multifunctional CD8^+^ T cell profile of up to four functions (IFN-γ, IL-2, perforin, and granzyme B). These findings suggest that the treatment induces an improved CD8^+^ T cell response in IND individuals, which could serve as a biomarker for monitoring the effectiveness of the anti-parasitic treatment (Mateus et al., 2017).

The long natural history of the disease makes difficult the monitoring of patients and the sequential study of the immune mechanisms underlying protection or disease pathogenesis. Indeed, experimental models of *T. cruzi* infection have been successfully used to propose or develop new strategies to combat the disease. Thereby, the second strategy was to investigate the existence of a correlation between the quality of the CD8^+^ T cell responses and the disease outcome in an animal model. For this, we established a murine model of acute (10 and 30 days) and chronic (100 and 260 days) Chagas disease using BALB/cAnNr 6-8 weeks old mice purchased from Charles River Laboratories International, Inc. (Wilmington, MA, USA). This model was characterized by parasite persistence for up to 260 post-infection days and moderate inflammation of the colon and liver in *T. cruzi*-infected mice. Chronically infected mice with moderate inflammatory infiltrate in liver tissue exhibited monofunctional antigen-specific cell responses along with high cytotoxic activity (granzyme B and perforin), and elevated levels of inhibitory receptors (predominantly CTLA-4 and PD-1) co-expressed on T cells. Taken together, these data support our previous results in chronically infected humans showing that *T. cruzi* persistence in mice promotes the dysfunctionality of T cells, and these changes might be correlated with Chagas disease progression (Mateus et al., 2019).

Once we established the murine animal model, we investigated the relationship between the infection outcome and immune T cell responses. First, we performed single infection experiments with DA (TcI) or Y (TcII) *T. cruzi* isolates. Second, because infections with diverse *T. cruzi* genotypes can occur in naturally infected individuals, mice were infected with the Y or DA strain and subsequently reinfected with the Y strain. We found differences in the infection outcomes in the two infection strategies used (Mateus et al., 2021). The single chronic infection displayed differences in the inflammatory infiltrate level, while mixed chronic infection by different *T. cruzi* DTUs showed dissimilarities in the parasite loads. Chronically infected mice with a low inflammatory infiltrate (DA-infected mice) or low parasitaemia and parasitism (Y/Y-infected mice) presented increases in early-differentiated CD8^+^ T cells, a multifunctional T- cell response and lower expression of inhibitory receptors on CD8^+^ T cells. In contrast, infected mice with a high inflammatory infiltrate (Y-infected mice) or high parasitaemia and parasitism (DA/Y-infected mice) showed a CD8^+^ T cell response distinguished by an increase in late-differentiated cells, a monofunctional response, and enhanced expression of inhibitory receptors. Overall, our results demonstrated that the infection caused by single or mixed *T. cruzi* infection with different parasite genotypes induce a differential immune CD8^+^ T cell response quality, which suggest that the quality of CD8^+^ T cell response might dictate differences in the parasite control and infection outcomes at the chronic *T. cruzi* stage (Mateus et al., 2017; Mateus et al., 2021).

## 5 Perspectives

The genetic variability of the parasite is an important issue to consider in studies on Chagas disease. In our studies, patients with Chagas disease were recruited according to the results of serological tests, and PCR tests were not performed to identify the presence or DTU of the parasite. Although all parasite DTU’s are known to be circulating in Colombia (Guhl and Ramirez, 2013), parasites belonging to DTU TcI and, to a lesser extent, DTU TcII, have been the ones mainly recovered from patients (Ramirez et al., 2010). In this context, our results from mice chronic infections with DTU TcI (DA), and DTU TcII (Y) strains showed that both parasite strain and type of infection (single or double) influenced parasitemia, parasitism and inflammatory response (Mateus et al., 2021). Therefore, it is possible that parasite DTUs could affect disease outcomes. However, further studies are needed to confirm this hypothesis.

In the same way, increasing the number of known genomes of different *T. cruzi*-DTU strains (Talavera-López et al., 2018; Gómez et al., 2020; Talavera-López et al., 2021), and their protein expression profiles during the amastigote and trypomastigote stages, is essential for the rational design of vaccine or immunotherapy strategies using specific-promiscuous epitopes expressed in the majority or almost all parasite strains. In this sense, based on the above-discussed characteristics of the TcTLE peptide, this sequence is an excellent candidate to be part of a subunit vaccine. Taking into account the experience provided by mRNA or attenuated adenovirus vector vaccines against SARS-CoV-2 and its advantages in the induction and production of immunity, this could be a platform to explore a potential Chagas vaccine (Park et al., 2021).

On the other hand, the advance in OMICS sciences constitutes a fundamental approach to translate the findings about the quality of CD8^+^ T cells immune response into strategies that help prevent the disease and make decisions in relation to the treatment, monitoring and management of patients at both the individual and collective level. A holistic approach to the components of the immune response in individuals with Chagas disease in different states of infection and disease is necessary. The comparative analysis of the immune response in an acute and chronic state of infection will allow a better understanding of the mechanisms involved in the early control of the infection and the factors that contribute to its progression and chronicity. Unfortunately, Chagas disease in most cases is diagnosed during the chronic phase, so the joint work of research groups is necessary to develop strategies for disease intervention. One study conducted by Gómez et al., 2021, assessed 106 immune markers expressed by parasite-stimulated PBMCs and compared them between HD and IND (Gómez et al., 2021). They found different expression patterns in 32 out of the studied genes. Thus, in IND patients, 23 upregulated genes were found, several of which were involved in immunological pathways such as antigen-dependent B-cell activation, stress induction of HSP regulation, NO_2_-dependent IL-12 pathway in NK cells, and cytokine-inflammatory response.

Based on the role of the innate immune response involved in the processing and presentation of parasite antigens to CD8^+^ T cells (Santander et al., 2007; Lasso et al., 2009; Acevedo et al., 2018), as a first approach, some studies on dendritic cells (DC) and monocytes, were conducted. For instance, our research group found that the immature DC of CCP, unlike those of HD, matured in the presence of *T. cruzi* HSP70 protein fragment producing a greater amount of the immunosuppressive cytokine IL-10 and less of IL-12, which polarizes the response towards Th1 lymphocytes (Cuellar et al., 2008). Similarly, we also reported that parasite antigens are capable of inducing a decrease in the expression of the costimulatory molecules CD40, CD80, and CD86 in DC, thus affecting the triggering of the T responses (Gómez-Olarte et al., 2020). As monocytes are heterogeneous and multifunctional cells, participating in both inflammation and healing in cardiovascular diseases, their total and subset populations were analysed. It was observed that CCP had a higher percentage of total monocytes (CD14^+^ CD16^+^), and total activated monocytes (HLA-DR^+^) than HD (Gómez-Olarte et al., 2019). It was also shown that the percentage of intermediate monocytes (CD14^++^ CD16^+^), which produce pro-inflammatory cytokines, was increased in CCC compared to IND and HD. Regarding classical or healing monocytes (CD14^++^ CD16^-^), they were increased in IND compared to CCC. Importantly, it was shown that in CCC, classical and intermediate monocytes were positively correlated with plasma levels of IL-6, a cytokine involved in the expansion of innate suppressor cells (Medina and Hartl, 2018). Together, these findings show the influence of the innate immune response on the development of the parasite-specific response during the natural course of Chagas disease.

Finally, some of our previously described findings (the decrease of CD8^+^ T cells proliferation capacity, CD28, and CD3ζ chain expression, and IL-2 production) are associated with suppression mechanisms mediated by innate suppressor cells derived from the myeloid line (Bronte et al., 2016). These suppressor cells derived from the myeloid line (MDSC) are characterized by a group of heterogeneous cells that have the ability to inhibit the activity of cells through mechanisms associated with the production of reactive oxygen species (ROS), expression of arginase 1 (ARG1) and inducible nitric oxide synthase (iNOS), and the presence of immunosuppressive cytokines (Medina and Hartl, 2018). Studies in mice have shown the importance of MDSCs in the infection control as well as and their future application in the development of immunotherapeutic measures against *T. cruzi* (Fresno and Gironès, 2021). Therefore, we are analysing MDSCs in patients with different stages of the disease to determine their role in human Chagas disease.

## 6 Conclusion

Given that the immune response and the equilibrium between its effector and its regulatory arms seem to be determinants in the outcome of chronic Chagas cardiomyopathy, we described a pipeline for exploring new candidate biomarkers for disease progression evaluation, treatment monitoring, and the development of epitope-based vaccine or immunotherapy strategies using OMICS and immunogenetics tools. Indeed, some of the alternatives that can be explored based on our findings are: (i) The inclusion of the TcTLE peptide in the rational design of epitope-based vaccines. (ii) The development of immunotherapy strategies that include the use of T_SCM_ or the blocking of inhibitory receptors. (iii) The discovery of new biomarkers based on IND and CCC transcriptomic comparisons of the molecules involved in the cellular signalling pathways implied in the correlation between the quality of the CD8^+^ T cell response and the outcome of the disease. (iv) The use of the CD8^+^ T cell response quality for monitoring treatments, immunotherapies or vaccines.

## Author contributions

CP conceptualized and wrote the draft of the manuscript. PL and JM designed and elaborated the figures and tables. CP, JG, PL, JM, and AC completed the literature search. CP, AC, and JG performed the funding acquisition. All authors contributed to the article and approved the submitted version.
